# Metagenomics and Bioinformatics in Microbial Ecology: Current Status and Beyond

**DOI:** 10.1264/jsme2.ME16024

**Published:** 2016-07-05

**Authors:** Satoshi Hiraoka, Ching-chia Yang, Wataru Iwasaki

**Affiliations:** 1 Department of Computational Biology and Medical Sciences, Graduate School of Frontier Sciences, the University of TokyoChiba 277–8568Japan; 2 Department of Biological Sciences, Graduate School of Science, the University of TokyoTokyo 113–0032Japan; 3 Atmosphere and Ocean Research Institute, the University of TokyoChiba 277–8564Japan

**Keywords:** metagenomics, bioinformatics, database, meta-analysis, long-read sequencer

## Abstract

Metagenomic approaches are now commonly used in microbial ecology to study microbial communities in more detail, including many strains that cannot be cultivated in the laboratory. Bioinformatic analyses make it possible to mine huge metagenomic datasets and discover general patterns that govern microbial ecosystems. However, the findings of typical metagenomic and bioinformatic analyses still do not completely describe the ecology and evolution of microbes in their environments. Most analyses still depend on straightforward sequence similarity searches against reference databases. We herein review the current state of metagenomics and bioinformatics in microbial ecology and discuss future directions for the field. New techniques will allow us to go beyond routine analyses and broaden our knowledge of microbial ecosystems. We need to enrich reference databases, promote platforms that enable meta- or comprehensive analyses of diverse metagenomic datasets, devise methods that utilize long-read sequence information, and develop more powerful bioinformatic methods to analyze data from diverse perspectives.

## Data deluge in microbial ecology

Although microbes play fundamental roles in various ecosystems, most have not yet been characterized in detail. Bioinformatics, which aims to discover new biological concepts and laws based on large-scale data, is now expected to accelerate discovery in unexamined areas of the microbial universe. The *data deluge* has made bioinformatics indispensable in modern research; recent innovative technologies are producing large amounts of data at an unprecedented pace. Observations are key to science; for example, optical and electron microscopies are important methods of observation combined with various staining methods. Among recent observational technologies, high-throughput DNA sequencing technologies have rapidly produced vast amounts of genetic information at low cost, making available thousands of microbial genomes. These genome sequences provide a comprehensive catalog of the microbial genetic elements underlying diverse microbial physiology, and also assist in weaving a massive tapestry of microbial evolutionary histories (72, 154).

In microbial ecology, research has been hindered because the majority of environmental microbes are uncultivable. A large number of studies across diverse natural environments have identified many microbial groups with no axenic culture (110, 113, 133, 138). In order to overcome this fundamental difficulty, culture-independent approaches, including DNA hybridization (*e.g.*, microarray and fluorescent *in situ* hybridization), DNA cloning, and PCR have been used to detect specific members and/or functional genes in microbial communities (3, 8, 9, 32, 34, 47, 56, 61, 74, 157, 174, 185). High-throughput sequencing technologies have recently popularized shotgun metagenomic and (typically 16S ribosomal RNA [rRNA] gene) amplicon sequencing methods, which identify members and/or functional genes at a greater scale and in more detail. Their use in diverse environments has revealed the presence of extremophiles (27, 33, 92), uncovered relationships between microbes and human diseases (10, 44, 55, 57, 85, 87, 96, 134, 147), and characterized the nutrition systems involved in symbiosis (68, 174, 177). Even more applications of these methods are used in agriculture (93), food science and pharmaceuticals (32), and forensics (49, 79, 82, 182). Many large-scale metagenomic projects are now generating comprehensive microbial sequence collections for different environments (*e.g.*, human-associated [116, 167], soil [54, 171], and ocean environments [17, 142]). Since microbial communities change as they interact with other organisms and as the environment changes, time-series analyses have also become common (21, 24, 77, 115, 172).

**Fig. 1:**
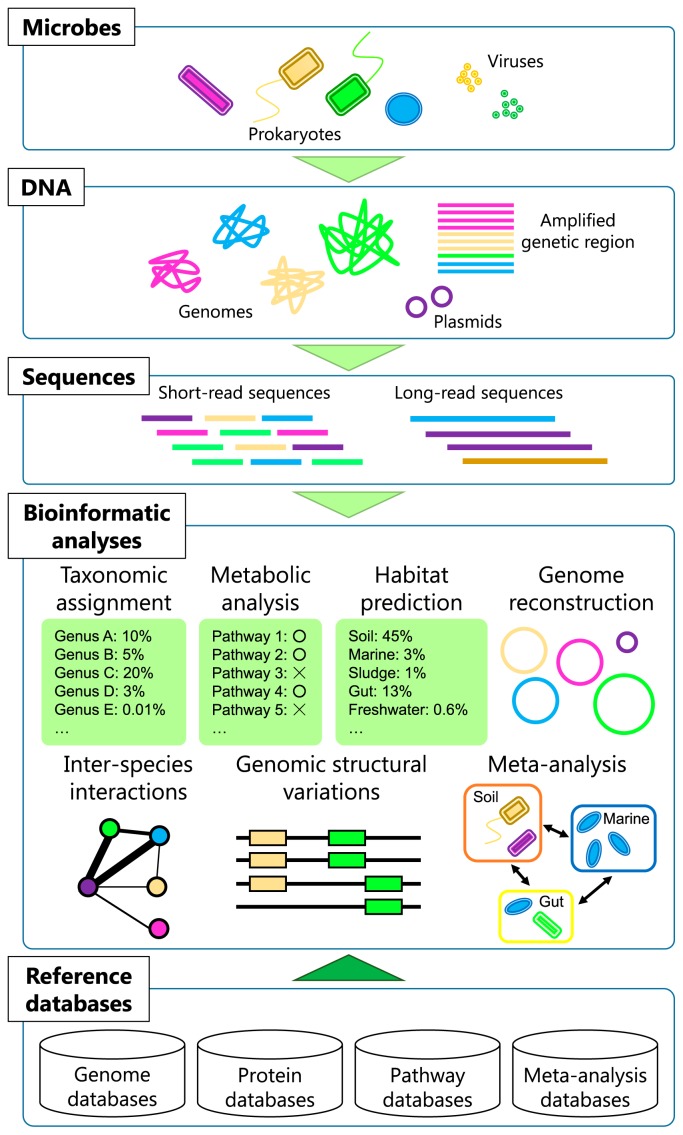
Schematic figure of metagenomic and bioinformatic analyses in microbial ecology. The illustration covers topics that are already popular, that need further development, and that will become important in the future. At the bottom of the illustration, reference databases lay foundations for various bioinformatic analyses.

Several bioinformatic tools have been developed and popularized to analyze metagenomic and amplicon sequence data. Web servers, such as MG-RAST (104), IMG/M (97), EBI Metagenomics (69), and SILVAngs (135), and pipelines, such as MEGAN (70), QIIME (25), and Mothur (145), now allow researchers to perform integrated metagenomic analyses and visualize results without command-line operations or strong computational knowledge. Since there are already several introductory articles on these popular tools (36, 88, 103, 123, 146), we herein addressed how can we examine large datasets in detail in order to obtain a deeper understanding of the ecology and evolution of microbes in the environment beyond existing approaches that are already popular (Fig. 1).

## Toward better taxonomic assignments

A fundamental step in microbial ecology is to describe the taxonomic distribution of microbial community members. Thus, the precise taxonomic assignment of sequencing reads is one of the most important issues in the analysis of metagenomic and amplicon sequencing data. Reference-based methods are frequently used for this purpose, in which taxonomic assignments are based on straightforward sequence similarity searches against reference genomes (*e.g.*, RefSeq [163]) or 16S rRNA sequence databases (*e.g.*, Greengenes [101], SILVA [135], RDP [31], and Ez-Taxon [29]). These databases typically contain the sequences of previously isolated and taxonomically classified strains, whereas they also contain environmental clone sequences. Many bioinformatic tools, such as TANGO (6), MetaPhlAn (148), and Kraken (176), have been developed to improve the computational efficiency, accuracy, and sensitivity of taxonomic assignments. Although these tools perform well for many applications, discriminating between closely related species is sometimes difficult, particularly in cases of highly conserved genes (*e.g.*, 16S rRNA genes). Additionally, genes that undergo horizontal gene transfer (HGT) between different taxa may cause incorrect taxonomic assignments. A more fundamental issue is taxonomic bias in reference databases, which leads to biased taxonomic assignments. A previous study reported that taxonomic assignments markedly change when different versions of reference databases are used (128). Therefore, even in this era of data deluge, the further taxonomic enrichment of reference databases is key to the improvement of reference-based methods. It is important to note that this issue is more crucial in the analysis of fungal and viral sequences because fewer reference sequences are available and their taxonomy is under debate. In order to overcome this obstacle, several projects are now attempting to obtain a number of genomic sequences to enrich databases (58, 180). In cases in which amplicon sequencing data are analyzed, the filtering of chimeric sequences formed during PCR is very important for precise analyses (63). Several bioinformatic tools, such as AmpliconNoise (136), ChimeraSlayer (63), and UCHIME (41), have been proposed and commonly used to remove chimeric sequences.

Reference-free methods may be used (*e.g.*, CD-HIT [50], UCLUST [40], and UPARSE [42]) as an alternative to reference-based methods. These methods use clustering to group marker genes, such as 16S rRNA, ribulose-1,5-bisphosphate carboxylase/oxygenase (RuBisCO), ammonia monooxygenase (*amoA*), sulfate thioesterase/thiohydrolase (*soxB*), and methyl-coenzyme M reductase genes (*mcrA*), into unique representative sequences that serve as operational taxonomic units (OTUs) (27). 16S rRNA genes are used to study the general composition of a microbial community, while RuBisCO, *amoA*, *soxB*, and *mcrA* genes are typically used to investigate microbes that play critical roles in carbon, nitrogen, sulfur, and methane cycles, respectively. In addition to traditional genes, useful marker genes may be found and used by comprehensively profiling metagenomic datasets (159). In reference-free methods, OTUs often cannot be assigned to known taxa. In order to estimate phylogenetic information for these OTUs, PhylOTU (150), pplacer (98), and PhyloSift (35) couple reference-free methods with phylogenetic analyses.

## Toward the cultivation-free reconstruction of genomic sequences

Most metagenomic studies currently focus at the level of individual genes (“gene-centric” metagenomics [132]). In contrast, in some pioneering research on “genome-centric” metagenomics, microbial genomes that include those of important uncultivated taxonomic groups were successfully reconstructed by metagenomic binning and assembly from various environments including oceans, groundwater, soil, hypersaline lakes, and acid mine drainage (4, 18, 45, 95, 112, 168). Although amplification bias still poses a non-negligible difficulty, single-cell genomic sequencing is expected to accelerate direct genome reconstruction from environmental samples (43, 90, 140), in which the combination of single cell genomic and metagenomic approaches may be a promising approach (102).

Metagenomic assembly is an important step for revealing the ecology and physiology of environmental microbes, in which the fundamental concepts of metagenomic assembly from short-read sequences have already been described in detail (36, 88, 103, 123, 146). Several tools have been developed for metagenomic assembly, and are classified into reference-based (*e.g.*, AMOS [130]) and *de novo* methods (*e.g.*, MetaVelvet-SL [2], SPAdes [119], and IDBA-UD [127]). In the case of *de novo* assembly, users need to consider chimeric contigs because similar genetic regions may be shared by different genomes (100, 129, 170). In order to improve the performance of *de novo* metagenomic assembly, composition-based methods use specific sequence features in a metagenomic dataset to split reads into different species. For example, CONCOCT (5), metaBAT (81), and MaxBin (178) bin sequences based on their tetra-nucleotide frequency composition and coverages. These composition-based approaches are computationally intensive, particularly in their memory usage. Thus, a fast-clustering approach using matrix decomposition with streaming singular value decomposition may be combined (30). On the other hand, sequence coverage information across different DNA extraction methods may also be used to effectively split sequences into species because the numbers of sequence reads from the same genome need to be similar regardless of the extraction method (4). A related approach bins co-abundant sequences across a series of metagenomic samples from similar environments (*e.g.*, human gut microbiome) to identify co-abundance gene groups (117).

Another information source that may improve the performance of metagenomic assembly is long-range contiguity. The recent development of methods to investigate long-range chromatin interactions (*e.g.*, Carbon-Copy Chromosome Conformation Capture [5C] [38] and Hi-C [11]) may also contribute to metagenomic assembly because these methods ligate sequences from two different genomic regions that are in the same cell (20). The Irys system (BioNano Genomics, San Diego, USA), which also detects long-range contiguity with fluorescently labeled DNA, may be used to obtain long contigs (64).

## Toward a more reliable estimation of community metabolism

Microbial genomes are affected by the environment during their evolution. Metabolic processes encoded in the genome, from biosynthesis to biodegradation, directly link microbial communities to the environment. Since most microbes are uncultivable, the direct estimation of community-scale metabolic pathways is also targeted by a metagenomic analysis. The most straightforward approach is to conduct sequence-similarity searches against pathway databases, such as KEGG (80), MetaCyc (22), and SEED (124), and use the findings obtained to annotate metabolic genes. Since many pathways with component genes that are only partially found in given metagenome data are typically detected with this naïve approach, MAPLE (160), MinPath (183), MetaNetSam (75), and HUMAnN (1) quantitatively or probabilistically evaluate whether these pathways likely function, enabling comparisons between samples. Significant biases in the databases of known pathways need to be taken into consideration when interpreting the findings of these methods. If shotgun metagenome data are unavailable, “virtual metagenomes” or functional gene abundance may be estimated using 16S rRNA amplicon sequencing data (89, 121). This approach takes advantage of closely related genomes being more likely to have a similar gene content, and, thus, given the 16S rRNA sequence, the gene content of its host genome may be estimated (at least, to some extent) if a closely related genome is already sequenced. It is important to note that such estimations may become difficult when applied to microbial groups with genomes that are rarely available and also that genomic variations within closely related microbial groups cannot be precisely considered. Despite these difficulties, this approach is very cost-effective and more easily applicable to large-scale comparative analyses.

## Toward a community-level analysis of genomic structural variations and dynamics

**Fig. 2:**
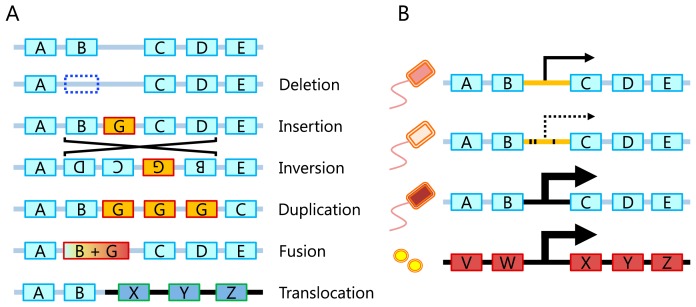
Schematic figures of genomic variations in environmental microbes. Each box represents a protein-coding gene, in which the letters indicate homology. Boxes and thick lines of different brightnesses represent genes and genomic fragments, respectively, that originated from different genomic areas or genomes. Dashed lines represent lost genes or expression. A: Types of genomic structural variations. B: Variations in regulatory sequences. Mutations (black dots) and the horizontal transfer of intergenic regulatory sequences (thick lines in black) both affect the strength of gene expression.

Operon structures, which are unique to prokaryotic genomes, reflect the function of their encoded genes and need to be associated with microbial ecological strategies. Thus, if we observe systematic variations in the gene order (or gene cluster structures) due to gene losses, fusions, duplications, inversions, translocations, and HGTs from an analysis of metagenome data, these variations may provide important clues for linking microbial communities to the environment (Fig. 2A). Although difficulties are sometimes associated with distinguishing variations under selection pressure from those because of population changes, MaryGold (118) is a tool for the visual inspection of such variations. Variations in the gene order for genes in the tryptophan pathway were identified within contigs assembled from the Sargasso Sea metagenome (78). Since the availability of long sequences that encompass multiple genes greatly facilitates a gene-order analysis, DNA cloning may also be used if the targeted pathways are efficiently enriched by colony selection (51, 158). On a larger scale, gene order may be affected by genome replication mechanisms. Most prokaryotic genomes are circular with one replication origin; thus, genes close to the origin may physically exist in multiple copies, particularly during an active growth phase. Thus, the detection of these regions from metagenomic sequences may reveal the growth dynamics of microbes in a community (86).

Among various sources of genomic variations and dynamics, HGT is of particular interest because it may help microbes adapt quickly to different environments (120, 139, 156). Although many comparative genomic studies have analyzed HGT (26, 73, 153), its role in microbial communities has not yet been elucidated in detail. A classical approach to detect HGT within communities is based on DNA cloning, which is particularly effective if colony selection is applied to the targeted genes (162, 175). In metagenomics, assembled contigs may be used to comprehensively identify HGT events by analyzing phylogenetic incongruence and gene order differences (62, 125, 161). In addition, gene gains via plasmids are also important driving forces that accelerate microbial adaptation to their environment. In accordance with this hypothesis, plasmids are frequently reported to contain genes that may contribute to fitness, such as detoxification genes (151, 152, 165) and antibiotic resistance genes (13). Notably, plasmid-specific metagenomics, or a *plasmidome* analysis, is now being conducted to directly investigate environmental plasmids without culturing or cloning (37, 173). For example, the bovine rumen plasmidome was previously reported to contain genes that may confer advantages to their hosts (19). Rat gut (76) and wastewater (149) plasmidomes have also been investigated.

In addition to genes, regulatory sequences in intergenic regions are related to the function of nearby genes. Thus, variations in the comprehensive set of regulatory sequences, or the regulome, for example, by promoter propagation, may play important roles in microbial adaptation (99, 122). We envision direct investigations of regulomes in microbial communities, or a “metaregulome” analysis, becoming an attractive research field as technical advances occur in long-read metagenomics. In a metaregulome analysis, variations and/or possible transfers of regulatory sequences, in addition to those of coding genes, may be identified from metagenomic datasets (Fig. 2B) (48, 122).

## Toward a comprehensive analysis of inter-species interactions

Inter-species interactions, including mutualism and parasitism, are of general interest in microbial ecology (16). Using abundant information from large-scale metagenomic datasets, co-occurrences (or anti-occurrences) among microbes, hosts, and/or viruses have been studied, and, for example, species interaction networks have been identified (12, 23, 28, 46, 53, 94, 114, 155, 164, 184). Recent large-scale projects include the *Tara* Oceans project, which revealed interactions among all three domains and viruses (17). Since environmental samples were revealed to contain *environmental DNA* shed from large organisms in addition to microbial DNA (107), a combinatorial analysis of microbial and environmental DNA is expected to accelerate the analysis of interactions between microbes and larger organisms.

The viral metagenome is called the *metavirome* or simply the *virome*. Viruses also play fundamental roles in ecosystems; therefore, a virome analysis is becoming an important field. To date, viral communities in hypersaline (143, 144) and human gut (105) environments have been extensively studied, and antibiotic viruses have also been of interest (108). A novel bacteriophage present in the majority of published human fecal metagenomes was recently reconstructed (39), and phage-bacteria ecological networks were suggested to protect gut microflora from antibiotic stress (108). Since viruses are classified into different types of DNA and RNA viruses, different approaches must be combined for comprehensive analyses (169). The use of targeted sequence capture techniques to efficiently increase the proportion of viral reads in metagenomic samples may also be considered (179). The largest limitation in bioinformatic analyses of viromes is insufficient reference genome data. Similarity searches using viral sequences often result in no significant hits, suggesting that there are many unknown viruses. In order to overcome this limitation, several bioinformatic tools have been developed and used for virome studies, such as ViromeScan (137) for taxonomy assignment and Metavir 2 (141) for viral genome reconstruction. Another difficulty is that in contrast to prokaryotes that have universal marker genes for a phylogenetic analysis (*i.e.*, 16S rRNA), there is no such gene for viral studies. An analysis of clustered regularly interspaced short palindromic repeats (CRISPRs) is a related emerging field because these repeats represent previous exposures to (or attacks from) viruses (15, 109, 131). CRISPRs are found in approximately 40% of bacteria and approximately 90% of archaea (59), and, thus, a metagenomic analysis of CRISPRs will contribute to advancing the field toward a comprehensive analysis of viral-microbial interactions.

## Toward a meta-analysis of metagenomes

Abundant metagenomic datasets containing dozens of terabytes of sequence data are currently found in the Short Read Archive database at NCBI, and its content is increasing daily (84). Whereas each metagenomic dataset provides a snapshot of the microbial community at the time of sampling, a comprehensive analysis (or *meta-analysis*) of many datasets is expected to reveal general patterns or laws that determine how microbes interact with their environments and how their genomes have been shaped. It is important to note that different datasets have been constructed with different experimental methods and conditions.

Regarding global correlations between environments and microbial genomes, correlations involving genomic GC contents (66) and genome sizes (14) have been reported. MetaMetaDB (181) was developed for a meta-analysis of different environments inhabited by a microbe and the factors that contribute to adaptation. This database may be used to predict all possible habitats of microbes by searching for the presence of microbes in metagenomic and 16S rRNA amplicon sequencing datasets derived from diverse environments. Given a metagenomic or 16S rRNA amplicon sequencing dataset, researchers may find environments with microbial community structures that are similar to that dataset using MetaMetaDB (181). A meta-analysis of metagenomic datasets was also performed to examine microbial adaptation to environments in terms of metabolic flexibility (52, 60) and to investigate specific functional genes that facilitate adaptation to extreme habitats, such as heavy metal resistance genes (65, 106) and salt-stress responsive genes (166). In a meta-analysis, associations were found between membrane protein variations and oceanographic variables in a global ocean sampling expedition (126). Microbial interactions between humans and the indoor environment have also been investigated (91).

## Toward metagenomics with long-read sequencers

Sequencers with the ability to produce long-read data are currently being developed, such as the PacBio RS II (Pacific Biosciences, Menlo Park, USA) and nanopore-based sequencers (Oxford Nanopore Technologies, Oxford, UK). Long reads are already contributing to many types of bioinformatic analyses, including the high-quality *de novo* assembly of bacterial and viral genomes (7, 67) and the detection of genomic structural variations, such as large-scale insertions/deletions or HGTs in microbial communities (71). Long reads are expected to be helpful for reconstructing genomes from metagenome data, directly observing genomic structural variations, and analyzing metaregulomes in various microbial communities. High-density microbial habitats, such as biofilms and gut communities, may be interesting targets because their genomic structures may be changed by the frequent exchange of genetic materials.

Long-read metagenomics will be an emerging field, but there are still limitations to be considered. Although PacBio RS II with P6-C4 chemistry may generate reads with an average length of approximately 15 kb, less than 50,000 reads are generated per SMART cell (*i.e.*, less than 1 Gb in each SMART cell). This throughput is markedly smaller than that of the so-called massively parallel sequencers (*e.g.*, approximately 15 Gb in each run of MiSeq [Illumina]) and may be insufficient for describing taxonomically diverse microbial communities. In addition, the low accuracy of PacBio RS II reads (approximately 85%) may hinder a bioinformatic analysis, unless highly redundant sequencing (*e.g.*, more than 50X coverage) is performed to reach high accuracy in the ensemble. Along with the development of new bioinformatic methods, protocols also need to be optimized to avoid DNA fragmentation during extraction (83, 111).

## Concluding remarks

Metagenomic and bioinformatic approaches are already common in microbial ecology and have been used to investigate whole communities containing many types of uncultivable microbes (Fig. 1). However, to date, most analyses have depended on straightforward sequence similarity searches against reference databases. This may not be satisfactory because microbial genomes need to be the fundamental basis for microbial ecology and evolution. The enrichment of reference sequences (for microbial taxa and functional genes) is one of the fundamental issues for promoting various kinds of analyses. Platforms that enable a meta-analysis of diverse metagenomic datasets will allow us to discover the hidden laws of the microbial ecosystem from publicly available data. Long-read sequence information will open up the possibility of studies that focus on subjects that have not yet been examined in detail by using short-read sequences. Furthermore, more powerful bioinformatic methods for analyzing data from diverse perspectives are required in order to advance past routine metagenomic analyses.
